# Optimization of Glass-Powder-Reinforced Recycled High-Density Polyethylene (rHDPE) Filament for Additive Manufacturing: Transforming Bottle Caps into Sound-Absorbing Material

**DOI:** 10.3390/polym16162324

**Published:** 2024-08-16

**Authors:** Sarah Iftin Atsani, Swee Leong Sing

**Affiliations:** Department of Mechanical Engineering, National University of Singapore, Singapore 117575, Singapore; e0922434@u.nus.edu

**Keywords:** additive manufacturing, 3D printing, recycling, life cycle analysis, fused deposition modeling

## Abstract

Additive manufacturing presents promising potential as a sustainable processing technology, notably through integrating post-consumer recycled polymers into production. This study investigated the recycling of high-density polyethylene (rHDPE) into 3D printing filament, achieved by the following optimal extrusion parameters: 180 °C temperature, 7 rpm speed, and 10% glass powder addition. The properties of the developed rHDPE filament were compared with those of commonly used FDM filaments such as acrylonitrile butadiene styrene (ABS) and polylactic acid (PLA) to benchmark the performance of rHDPE against well-established materials in the 3D printing industry, providing a practical perspective for potential users. The resulting filament boasted an average tensile strength of 25.52 MPa, slightly exceeding ABS (25.41 MPa) and comparable to PLA (28.55 MPa). Despite diameter fluctuations, the filament proved usable in 3D printing. Mechanical tests compared the rHPDE filament 3D printed objects with ABS and PLA, showing lower strength but exceptional ductility and flexibility, along with superior sound absorption. A life cycle analysis underscored the sustainability advantages of rHDPE, reducing environmental impact compared to conventional disposal methods. While rHDPE falls behind in mechanical strength against virgin filaments, its unique attributes and sustainability position it as a valuable option for 3D printing, showcasing recycled materials’ potential in sustainable innovation.

## 1. Introduction

In recent years, the global production of plastics has experienced a significant surge. As per the data provided by the Statista Research Department [1], the yearly global production of plastics surpassed 390.7 million metric tons in 2021, marking an annual growth rate of 4% and indicating a continuing upward trend. However, the escalating production of plastics has been accompanied by environmental issues, such as the build-up of plastic waste in land and ocean ecosystems. An estimated range of 4.8–12.7 million tons of plastic waste is discarded into the world’s oceans on an annual basis [2], resulting in severe marine contamination and constituting a substantial hazard to marine organisms. Furthermore, it is emphasized that of the 6.3 billion metric tons of plastic waste generated, a shocking proportion of 79% has piled up in landfills or the natural environment [3].

As the magnitude of plastic production persists on its upward trend, there is an increasing necessity to mitigate the intricate environmental repercussions. Landfilling, as a temporary measure for plastic waste, remains inadequate in addressing the escalating plastic production and minimal recycling rates [4]. Hence, there is an urgent need for plastic recycling technologies. Beyond existing methods, emerging technologies such as additive manufacturing (AM) using recycled polymers could be an innovative approach to reduce plastic waste.

AM, often known as 3D printing, has emerged as a revolutionary technology for producing functional components across various fields [5]. Its ability to create complex geometries with high precision has positioned it as a preferred method for modern manufacturing [6]. One of the key advantages of AM is its potential as a clean processing technology, especially when integrating post-consumer recycled polymers into the production cycle [7]. This approach not only leverages the principles of the circular economy but also offers a significant solution to the escalating issue of global plastic contamination.

Research by Beltrán et al. [8] explored the recycling potential of polylactic acid (PLA) from 3D printing waste. By examining the properties of recycled PLA film, the findings suggest that adopting a distributed recycling approach, which focuses on a consistent and well-characterized PLA grade, could yield mechanically robust recycled materials that remain compostable at the end of their lifecycle [8]. Additionally, the study by Mishra et al. [9] involved repurposing acrylonitrile butadiene styrene (ABS) waste plastic from post-industrial sources by blending it with virgin ABS pellets. These mixtures were extruded into filaments and demonstrated successful application in 3D printing through the fused deposition modeling (FDM) technique [9]. Additionally, the efforts of Herianto et al. demonstrated promising results in recycling polypropylene waste into 3D printing filament using material extrusion [10]. Similarly, research by Arrigo et al. successfully created a 3D printing filament by recycling polypropylene from urban waste and reinforcing it with talc [11]. Further, Daliri et al. explored the potential of recycling polypropylene from mushroom boxes into 3D printing filament by mixing it with glass fiber, showcasing the versatility and viability of polymer recycling in additive manufacturing [12].

However, efforts to recycle polymers into 3D printing filaments have predominantly focused on common materials such as PLA [13,14,15,16,17] and ABS [18,19,20,21,22]. These materials are well-studied and widely used in the 3D printing industry. However, there is a notable gap in research concerning high-density polyethylene (HDPE), despite it being a major contributor to plastic waste [23]. Addressing this gap is crucial, as HDPE constitutes a significant portion of discarded plastics, particularly in the form of disposable water bottle caps [24].

HDPE is a type of polyolefin polymer with only 5 to 10 short-chain branches per 1000 carbon atoms [25]. As a semicrystalline polymer, when cooling from a molten state, HDPE often exhibits significant shrinkage due to the long-range ordering of polymeric chains [25]. This material also suffers from considerable warpage and poor adhesion to the print bed, which can lead to print failures [25]. Hence, advancing the creation of polyolefin filaments is crucial to enhance their versatility in design and manufacturing. While HDPE is usually processed using various common melt-processing methods such as injection molding and extrusion, the feasibility of recycling HDPE for use in AM has been documented, for instance, by Daniele et al. [26]. The study evaluated the printability and mechanical performance of recycled HDPE, highlighting both the challenges and opportunities associated with its use in AM.

However, the majority of the research indicates that recycled filaments often show reduced mechanical properties [27,28,29,30,31,32]. For instance, a study by Charles et al. found that the mechanical strength of recycled ABS polymer decreases with each recycling cycle [33]. Tensile tests revealed a gradual decline in maximum tensile stress and elastic modulus with successive recycling. While the initial drop in tensile stress from the original filament to the first recycled one is minimal, more substantial reductions occur in the second and third cycles. Specifically, Young’s modulus drops by 25% in the second cycle and 16% in the third cycle compared to the virgin material [33]. The reduction in mechanical properties can be attributed to the chain scission of polymeric chains, leading to progressive embrittlement of the recycled material [15]. Additionally, factors such as a reduction in chain length, lower molecular weight, and increased crystallinity collectively promote crack propagation [15].

Therefore, enhancing the mechanical behavior of recycled filament is a key focus in recent research. One promising approach is the addition of additives to the base material. For example, Kristiawan et al. studied the effect of incorporating glass powder into recycled polypropylene (rPP) from food packaging for FDM filaments [34]. Their results indicated that adding glass powder enhanced thermal stability and increased the melting temperature of the rPP. Furthermore, specimens with 10% glass powder showed significant improvements, with a 38% increase in ultimate tensile strength and a 42% rise in Young’s modulus compared to pure PP specimens [34].

The present research aims to explore the properties of 3D printing extrusion using rHDPE derived from disposable water bottle caps. To enhance the material properties, different percentages of glass powder were incorporated into the HDPE matrix. This study focuses on validating the filament as a viable feedstock for commercial 3D printing extrusion equipment. The quality of the 3D printed objects was assessed through detailed microstructure observations and by comparing the mechanical properties of the printed specimens with those of commercially available 3D printable materials. Additionally, in this study, the potential application of rHDPE in sound absorption was explored, comparing its performance with commercially available materials. This aspect of the study opens new avenues for the utilization of rHDPE, particularly in applications where sound absorption is critical.

Finally, this investigation will not only advance the understanding of rHDPE’s potential in AM but also contribute to sustainable practices by promoting the use of recycled materials. By extending the life cycle of HDPE through its application in 3D printing and exploring its sound absorption capabilities, this research underscores the pivotal role of innovative recycling strategies in mitigating plastic pollution.

## 2. Materials and Methods

### 2.1. Materials

The rHDPE material utilized in this study was sourced from used water bottle caps. To prepare the material, the bottle caps underwent a thorough washing process to remove dirt and surface impurities. Subsequently, they were dried using sunlight exposure. Once completely dry, the caps were shredded into flakes with an average particle size of 5 mm using a shredder machine operating at 200 rpm. The glass powder, sourced from crushed glass and sieved through a mesh size of 200, was used for reinforcement. The rHDPE flakes were physically mixed with glass powder (GP) in various weight ratios, specifically rHDPE/GP at 100/0, 90/10, and 80/20.

### 2.2. Filament Extrusion

The extrusion process was carried out using a single screw extruder by Felfil Evo. Prior to initiating extrusion, the extruder barrel was preheated to 180 °C for 15 min to eliminate any trapped moisture within the setup. During the actual extrusion, the temperature was adjusted to various levels, ranging from 180 °C to 220 °C. Inside the extruder barrel, the material underwent softening and liquefaction. The softened material was then forced out through a 1.75 mm-diameter nozzle, which was then cooled down by an air fan. Table 1 shows the extrusion parameters used in this study.

The design of the experiment employed the Taguchi method with an L9 orthogonal array, and each configuration (Table 2) was repeated three times. The observed output was the tensile strength of the filament. In this project, the L9 orthogonal array accommodated three factors with three levels of parameters (as detailed in Table 1). This design allowed for independent factor evaluation while minimizing the number of trials required. Once data were collected from the arrays, Taguchi analysis was conducted to guide the selection of parameter values to optimize the filament’s performance.

### 2.3. Differential Scanning Calorimetry (DSC)

A DSC experiment was performed to determine the melting point and degree of crystallinity of the polymer. The melting point illustrates the temperature span during which the polymer shifts from being rigid and somewhat brittle, akin to glass, to becoming soft and rubbery, while the degree of crystallinity describes the proportion of a material that has a crystalline structure. The degree of crystallinity ($X_{c}$) can be measured by calculating the ratio of the crystalline part of the semicrystalline polymer, shown in Equation (1). The experiment utilized a Mettler Toledo DSC1 apparatus, operating from 0 to 250 °C with a heating rate of 10 °C/min under ambient air conditions.

(1) $$X_{c}=\frac{\Delta H_{m}}{\Delta H_{100}}\times \frac{100}{100-w_{GP}}$$

where $\Delta H_{m}$ represents the heat of fusion of the sample, which is calculated from the area under the endothermic peak in DSC analysis. $\Delta H_{100}$ stands for the heat of fusion of a fully crystalline material—in this case PE, with a value of 293 J/g [35]—and $w_{GP}$ is the weight ratio of glass powder in the mixture.

### 2.4. Fused Deposition Modeling (FDM)

To fabricate the 3D printed specimens, an FDM commercial 3D printer CUBICON SinglePlus with a 0.4 mm nozzle was used. The rHDPE filament was compared to the commercial ABS and PLA filaments. The commercial filaments used were ABS-A100 and PLA-i21, both from CUBICON. The 3D printing parameters for the PLA and ABS material were based on their optimal parameters as adapted from [36,37]. Table 3, Table 4 and Table 5 show the printing parameters for ABS, PLA, and rHDPE filament, respectively.

### 2.5. Tensile Test

**Figure 1:**
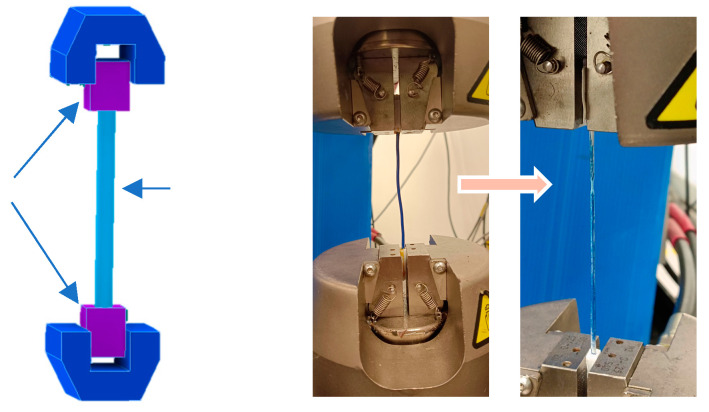
Tensile test method for filament.

The tensile tests were performed using a universal testing machine, Instron 8874. Tensile tests were conducted for both filaments and 3D printed specimens. The filament with a length of 110 mm was clamped between the 3D printed gripper, which was attached on both tips of the filament (Figure 1). The gripper was used to distribute the clamping force on the filament. The tensile tests were conducted with a 20 kN load and 5 mm/min speed until necking and stretching of the filament. Meanwhile, the 3D printed specimen followed the ASTM D638 standard [38]. The dog bone specimen was tested with a 20 kN load and 5 mm/min speed until the specimen failed.

### 2.6. Flexural Test

**Figure 2:**
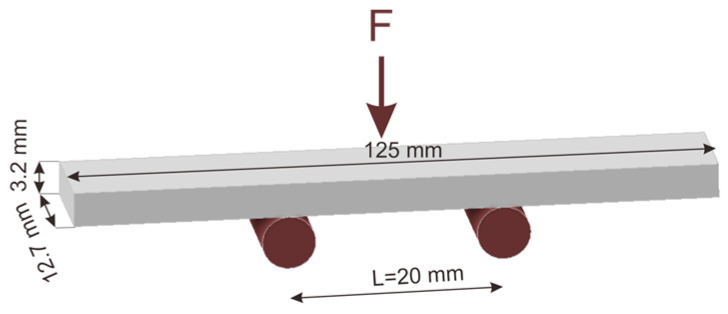
Flexural test for 3D printed specimen method.

Flexural experiments were performed on 3D printed samples of 125 mm × 12.7 mm × 3.2 mm by following the ASTM D790 standard [39]. The specimens were loaded in a three-point bending grip mounted on an Instron 8874 universal testing machine. The span between the two supports was L = 20 mm (Figure 2). Tests were performed with a 20 kN load, and crosshead speed of 2 mm/min, and the test ended at a displacement of 4 mm.

### 2.7. Compression Test

The compression tests were performed on 3D printed cylinders using a Shimadzu SFL-25AG machine by following the ASTM D695 standard [40]. The specimens were tested with 20 kN load at 2 mm/min speed until the specimens failed.

### 2.8. Printed Part Quality

The final quality assessment of 3D printed specimens was conducted through microscopic observations and surface roughness measurements. The examination of the surface morphology and structural details of the printed samples was performed using the Keyence VHX−6000 digital microscope equipped with a magnification of 20 to 200 times. Additionally, scanning electron microscopy (SEM) was employed to further inspect the surface topography of the specimens. Meanwhile, to quantify the surface smoothness, surface roughness measurements were carried out using a Mitutoyo SJ−210 stylus profiler on 6 different points across the printed specimens.

### 2.9. Sound Absorption Test

The sound absorption test was conducted using the BSWA SW4601 series impedance tube to determine the absorption coefficient, adhering to the ISO 10534–2 standards. The investigation was conducted in a high frequency range, spanning from 500 Hz to 6400 Hz.

**Figure 3:**
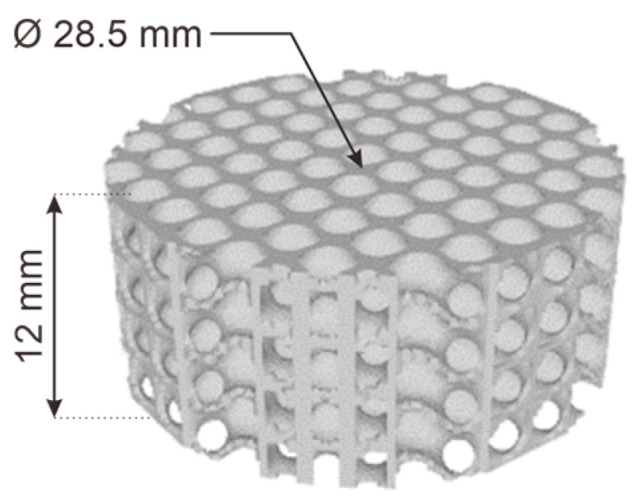
Sound absorption sample design.

The samples for the sound absorption test were cellular diamond structures designed using MSLattice software. The sample design (Figure 3) was a cylindrical shape with a 12 mm height and 28.5 mm diameter to fit the dimensions of the experiment apparatus. The structure had a 6 mm unit cell, 30% relative density, and 20% mesh.

### 2.10. Environmental Implications

**Figure 4:**
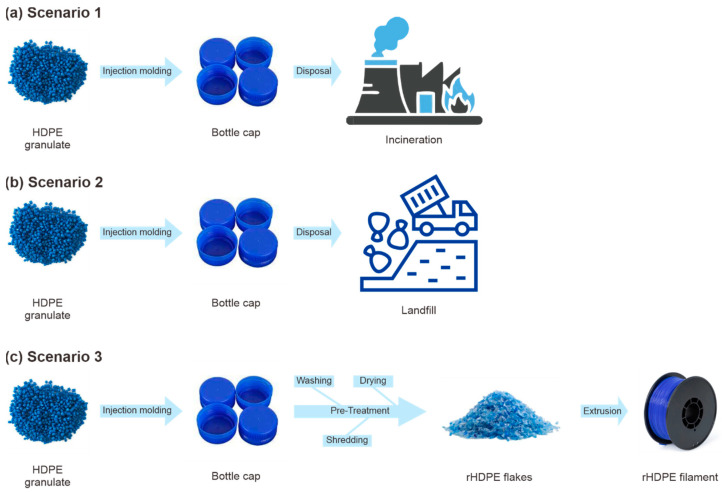
Life cycle flow of (**a**) Scenario 1; (**b**) Scenario 2; (**c**) Scenario 3.

The software used to perform the life cycle analysis was OpenLCA 2.0.2. For this study, the database used was the ELCD (European Reference Life Cycle Database) version 3.2. ReCiPe 2016 Endpoint Hierarchist version was selected as the methodology for calculating environmental impacts. The ReCiPe method is widely utilized in various research domains, particularly for life cycle analyses related to AM from a hierarchical cultural perspective. There are three different scenarios to compare, each with its flow details as follows:

1. Scenario 1: Cradle to incineration (Figure 4a). Scenario 1 begins with the production of bottle caps at the factory. The raw material used for producing bottle caps is high-density polyethylene granulate (HDPE), a production mix, at the plant. The material is injection-molded into bottle cap form. The used bottle caps are then disposed of in the incineration plant.
2. Scenario 2: Cradle to landfill (Figure 4b). Scenario 2 also begins with the production of bottle caps at the factory with the similar raw material of HDPE granule, which is injection-molded into the bottle cap. In this scenario, the used bottle caps are disposed of in the landfill.
3. Scenario 3: Cradle to filament (Figure 4c). Like the two previous scenarios, Scenario 3 also begins with the production of bottle caps. The difference is that the used bottle caps are not disposed of but recycled instead. The used bottle caps are collected and undergo pre-treatment processes such as washing and drying, then shredded into flakes. After that, the plastic flakes are extruded into filament by the extrusion machine.

In this study, the transporting, packaging, and use phases were not considered since the environmental impact comparison was mainly focused on the production and end-of-life phase.

## 3. Results and Discussion

### 3.1. Filament Quality

HDPE is a semicrystalline polymer that undergoes significant volumetric contraction during crystallization [41]. This leads to high residual stress levels within printed parts, causing warpage and shrinkage [41]. As HDPE is extruded through a nozzle, its viscosity decreases sharply, but it increases significantly upon solidification on the print bed [41]. Adding glass powder to HDPE increases the composite’s viscosity during extrusion and changes its flow behavior. The rigid particles in the polymer matrix restrict polymer chain mobility, resulting in higher viscosity [42]. The minimal viscosity changes during and after exiting the nozzle can enhance dimensional stability and minimize warping, though it may necessitate higher extrusion pressures.

Furthermore, when the polymer exits the nozzle, it transitions from confined flow to a free surface, typically causing an increase in diameter known as die swell [43]. Research by Mehrjerdi et al. indicates that adding filler material can reduce this swelling [44]. Die swell can also be minimized by raising the extrusion temperature [41]. The chosen filament extrusion temperature in this study ranges from 180 to 220 °C, which is above the melting temperature, and ensures that the rHDPE is adequately melted and can flow through the nozzle with minimal die swell.

**Figure 5:**
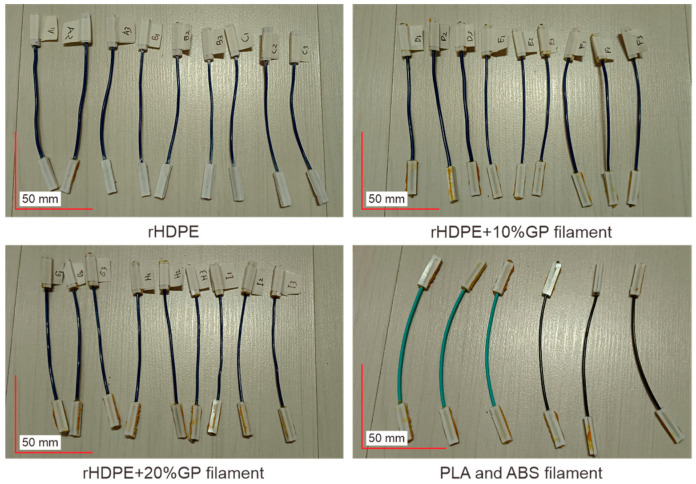
Filaments for tensile test.

Figure 5 illustrates the filaments produced through the extrusion process, which are set to be subjected to a tensile test. Table 6 provides the tensile properties of each rHDPE filament in comparison with the commercial filaments ABS and PLA.

**Figure 6:**
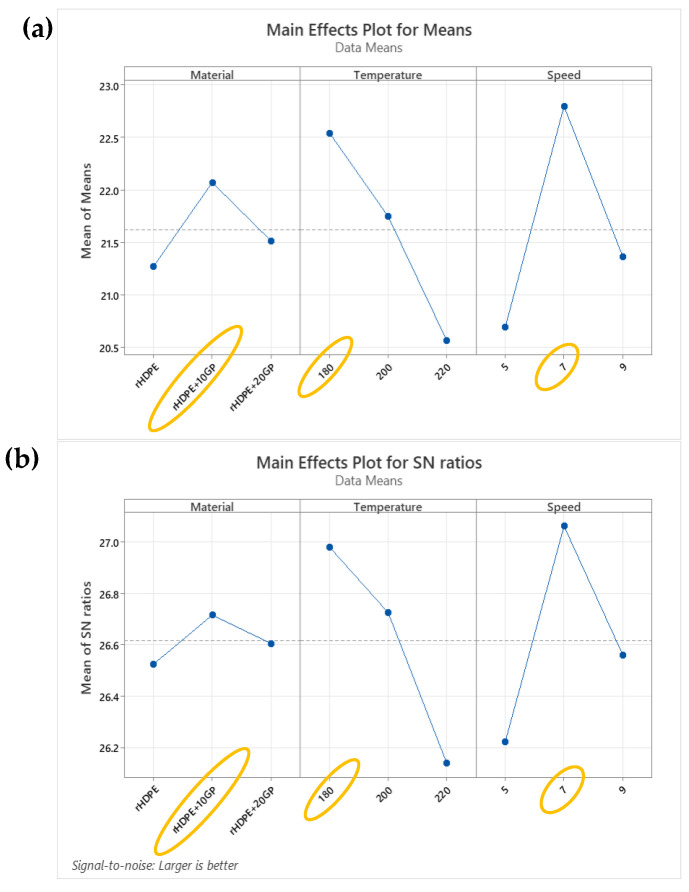
Taguchi analysis results: (**a**) main effects plot for means; (**b**) main effects plot for S/N ratio.

A statistical analysis was conducted utilizing the Taguchi method to ascertain the optimal parameters. The primary data output in this investigation is the tensile strength; hence, the signal-to-noise (S/N) ratio was computed using the “larger is better” characteristic. Figure 6 indicates that the optimal parameters for achieving the highest tensile strength in the filament are the use of rHDPE + 10%GP material, an extrusion temperature of 180 °C, and an extrusion speed of 7 rpm. Therefore, these parameters were employed to extrude the filament that was utilized for 3D printing specimens.

The average UTS of the rHDPE + 10%GP filament with its optimal parameters was 25.52 MPa. This value is quite competitive when compared to the two commercial filaments, indicating its promising potential. It is slightly more than the average UTS of ABS filament, which is 25.41 MPa, but it is lower than the average UTS of PLA filament, which stands at 28.55 MPa.

**Figure 7:**
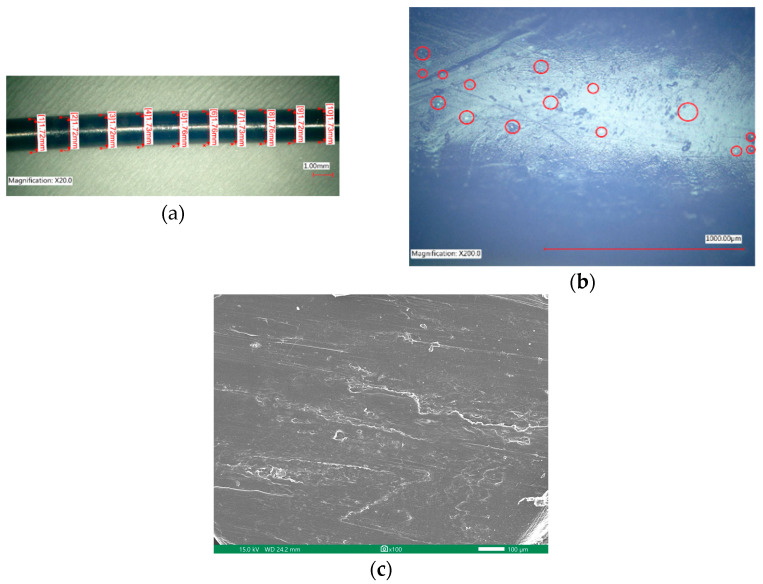
Filament observation under microscope: (**a**) diameter of the filament; (**b**) surface of the filament; (**c**) SEM image of the filament.

The diameter of the extruded rHDPE filament is not uniform; it exhibits some fluctuations. However, the diameter is within a range of 1.5 mm to 1.8 mm. This range is acceptable as it allows the filament to be inserted into the 3D printer’s nozzle. Figure 7a presents a measurement of a small portion of the filament, revealing that even within this small segment, the filament’s dimensions are not constant. Figure 7b provides a magnified view of the filament’s surface. It is notably smooth, indicating good quality, and potentially contributes to successful 3D printing outcomes. Furthermore, the filament contains a certain amount of glass powder, as indicated by the area within the red circles in Figure 7b. Figure 7c shows the SEM image of the cross-section area of the rHDPE filament created.

### 3.2. Differential Scanning Calorimetry (DSC)

**Figure 8:**
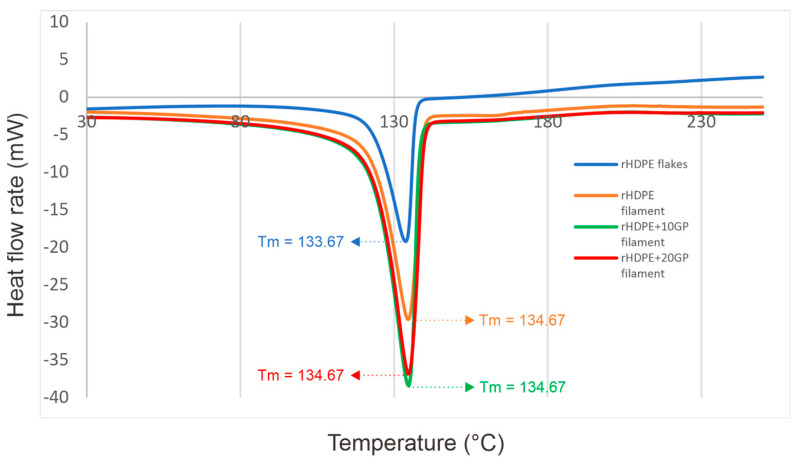
DSC curves of specimens.

To assess the impact of incorporating glass powder into the rHDPE matrix and to detect any potential degradation effects resulting from the extrusion process, differential scanning calorimetry (DSC) was employed. The DSC curves for all specimens are depicted in Figure 8, which includes the derived thermal characteristics such as the melting point. As per the figure, the melting point of all rHDPE filaments was observed to be 134.67 °C, marginally exceeding the melting point of rHDPE flakes, which was 133.67 °C. No significant variation was discernible between the rHDPE filament, the rHDPE + 10GP filament, and the rHDPE + 20GP filament.

Table 7 provides a detailed breakdown of the degree of crystallinity for each sample. As observed in Table 7, the crystallinity varied among the four samples. The rHDPE filament exhibited the least crystallinity, even less than that of the rHDPE flakes. Meanwhile, the greatest crystallinity was shown by the rHDPE + 20GP filament, followed by the rHDPE + 10GP filament.

Incorporating glass powder into the rHDPE matrix did not alter the blend’s melting point. However, the increase in glass powder content did enhance the blend’s degree of crystallinity (X_c_). This suggests that, when present in certain amounts, glass powder could serve as nucleation sites, accelerating the composite’s crystallization process. However, a higher degree of crystallinity is not beneficial for 3D printing as it could lead to increased warping during the printing process [45].

The table also reveals that the rHDPE filament’s melting point was higher than that of the rHDPE flakes. Additionally, both the heat of fusion and the degree of crystallinity were lower for the rHDPE filament compared to the rHDPE flakes. This suggests that the extrusion process employed in this study does not deteriorate the thermal characteristics of rHDPE. However, if the process is repeated more than once, it could potentially impact these properties, as noted in a separate study by Dacosta et al. [46].

### 3.3. Tensile Properties of 3D Printed Specimens

**Figure 9:**
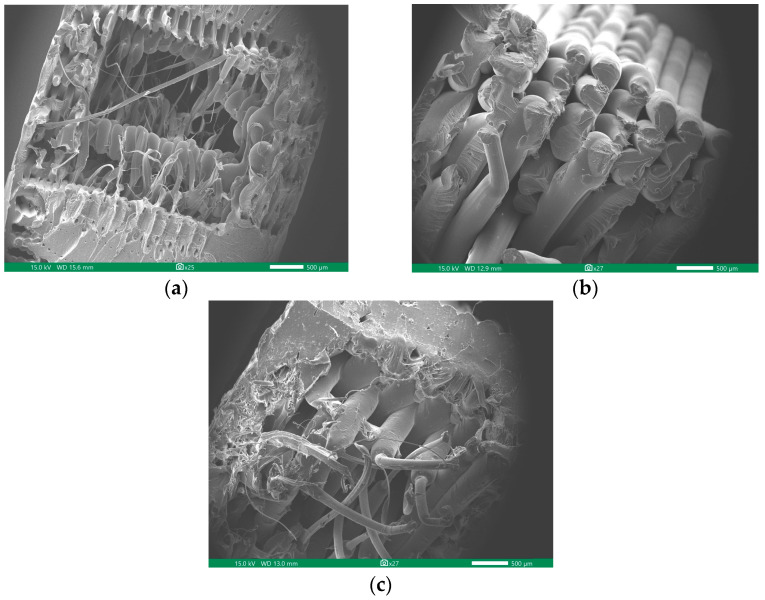
SEM image of cross-sectional fracture surface of (**a**) PLA; (**b**) ABS; (**c**) rHDPE + 10%GP.

The data presented in Table 8 reveal notable disparities in the tensile properties among the tested materials. The PLA specimens exhibited superior characteristics, boasting an ultimate tensile strength (UTS) of 28.57 MPa, a yield strength of 27 MPa, and Young’s modulus of 1008.8 MPa. Following closely behind, ABS demonstrated respectable performance, with a UTS of 26.93 MPa, a yield strength of 24.86 MPa, and Young’s modulus of 965.26 MPa. In contrast, the rHDPE + 10%GP specimens showed inferior tensile properties, with UTS values 51% lower than that of PLA. Furthermore, both yield strength and Young’s modulus were significantly lower, measuring 66% and 72% less than PLA’s corresponding values, respectively. However, despite having lower tensile strength, rHDPE + 10%GP stood out in terms of elongation at break. This indicates greater ductility and flexibility, which can be advantageous in certain applications. Figure 9 illustrates the cross-sectional area of the tensile specimen’s fracture surface.

### 3.4. Flexural Properties of 3D Printed Specimens

Table 9 indicates that PLA exhibited good flexural strength properties, with the highest modulus of flexural (1802.33 MPa) and yield strength (52.73 MPa) compared to the other materials. However, its strain at break was relatively low, suggesting brittleness. ABS surpassed PLA in terms of ultimate flexural strength (UFS), indicating its ability to withstand bending forces. It showed a UFS of 66.13 MPa. ABS also exhibited better ductility than PLA, as evidenced by a higher strain at break (21.2%). Contrary, rHDPE + 10GP had the lowest strength properties among the three materials. Its UFS and yield strength were significantly lower (41.15 MPa and 20.63 MPa, respectively). However, rHDPE + 10GP compensated with the highest strain at break (22.97%), indicating the best ductility.

### 3.5. Compression Properties of 3D Printed Specimens

The data listed in Table 10 show that ABS had the highest ultimate compressive strength (UCS) at 81.31 MPa, making it the most robust material when subjected to compression. PLA exhibited the highest yield strength at 25.97 MPa and elastic modulus at 1163.9 MPa, suggesting good resistance to plastic deformation and remarkable stiffness. In contrast, rHDPE + 10GP showed the lowest UCS among the three materials at 22.52 MPa, accompanied by a relatively low yield strength of 6.86 MPa, indicating early plastic deformation. Furthermore, it demonstrated inferior stiffness compared to ABS and PLA (with an elastic modulus of 196.35 MPa), while its compressibility fell within a moderate range (%strain at break around 48.14%), denoting its ductility and flexibility characteristics.

### 3.6. 3D Printed Part Quality

While the 3D printing parameters for ABS and PLA were based on existing studies in [36,37], respectively, the 3D printing parameters for the rHDPE filament were determined through preliminary trials to ensure printability. According to Duty et al., a material is considered printable if it fulfills four requirements: it must be extrudable through a nozzle, retain its shape, bridge a gap of a specific length, and show geometric stability after cooling down to room temperature [47].

**Figure 10:**
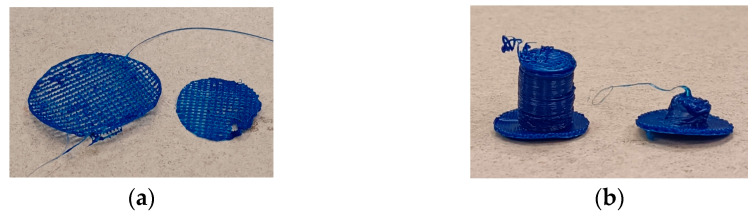
Trial-and-error stage of determining printing parameters of rHDPE: (**a**) discontinued layer due to undeposited filament from the nozzle; (**b**) material cannot retain its shape due to lack of cooling.

Firstly, to ensure the filament can be extruded through the nozzle, the nozzle temperature is adjusted. Once the fine temperature is found, the bed temperature is set to ensure that the deposited filament adheres to the build platform. The printing speed is also fine-tuned to ensure continuous deposition without interruptions. The fan speed is adjusted to keep the newly deposited filament in a molten state, allowing it to adhere to the previously printed layers. Additionally, a brim is added to the parameters to help the printed part adhere firmly to the build platform. Figure 10 shows some failures encountered during the trial-and-error process of determining the printing parameters of the rHDPE filament created in this study.

The four criteria of printability mentioned earlier are in fact influenced by the rheological properties of the polymers. Especially in the extrusion process, the viscosity and shear modulus of the melt polymer are critically important [41]. Rheological properties are intimately linked to a polymer’s physical traits and can predict how it will perform during processing [48]. On top of that, while achieving high printing speeds is economically beneficial, maintaining good quality of the 3D printed parts is equally important [48]. Understanding how polymers behave under high shear rates from rapid printing speeds can help enhance productivity, lower defects, and cut production costs [48]. Additionally, the rheological properties of polymers influence both the shape and mechanical characteristics of 3D printed parts [49]. Understanding these properties can assist better in adjusting printing parameters to achieve the desired final qualities.

**Figure 11:**
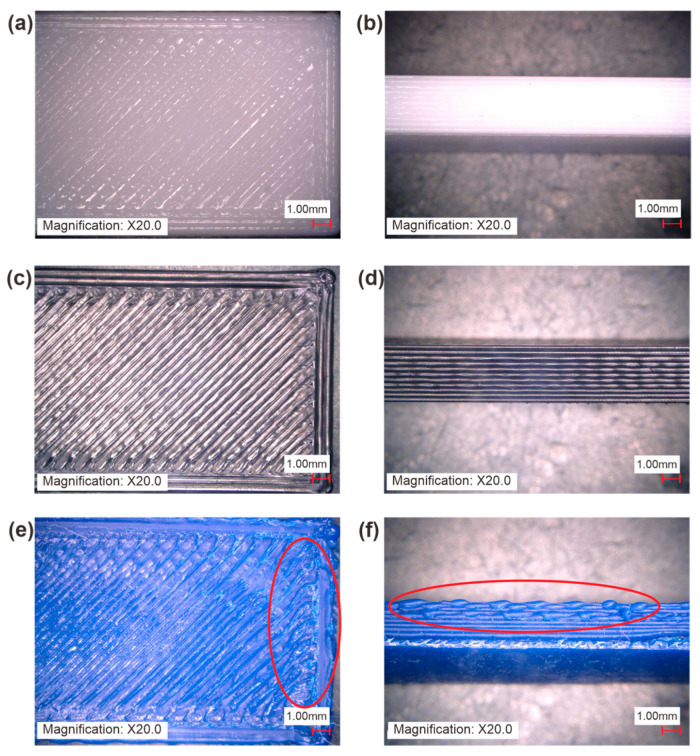
Microscope observation: (**a**) PLA top view; (**b**) PLA front side view; (**c**) ABS top view; (**d**) ABS front side view; (**e**) rHDPE + 10%GP top view; (**f**) rHDPE + 10%GP front side view.

**Figure 12:**
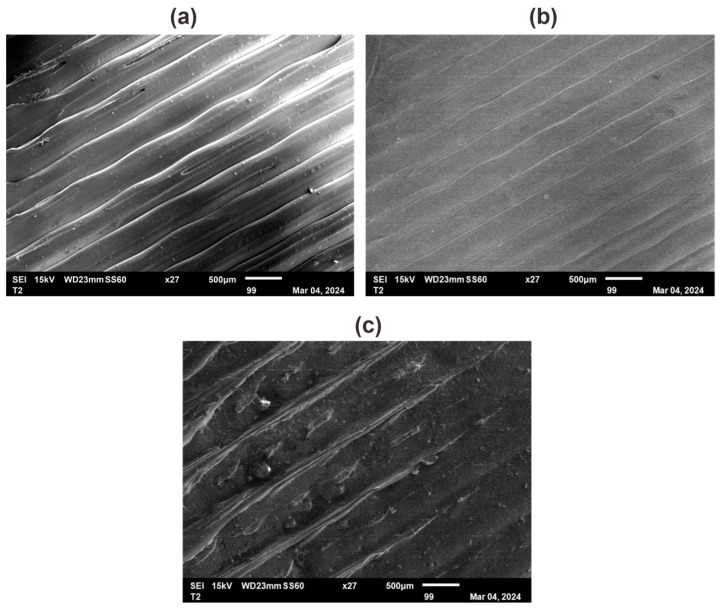
SEM observation: (**a**) PLA; (**b**) ABS; (**c**) rHDPE + 10%GP.

In terms of surface quality, surface roughness analyses were conducted to investigate the discerning differences between PLA, ABS, and rHDPE + 10%GP. These analyses were performed after printing all materials by using consistent parameters, including a layer height of 0.3 mm, an infill density of 50%, and a linear infill pattern. The findings presented in Table 11 reveal that specimens printed with rHDPE filaments exhibited higher Ra values compared to those made from virgin ABS and PLA filaments. This higher Ra value indicates a rougher surface texture. Notably, the top surface of the rHDPE + 10%GP specimen appeared particularly untidy, especially at the corners (see Figure 11e). Additionally, the front side view of the rHDPE + 10%GP specimen (Figure 11f) indicated a degradation in printing layer quality toward the top. This phenomenon may be attributed to non-uniform material deposition during the printing process of the rHDPE + 10%GP filament, as depicted in Figure 12c.

The inconsistency in extrusion quality also contributes to the reduced mechanical strength [50] observed in the rHDPE specimens compared to PLA and ABS due to several factors. One possible factor is poor filament quality [51]. Recycled plastic may contain impurities or small particles that are extruded together with the main material, resulting in a low filament quality [52]. Additionally, unevenly mixing glass powder with the polymer matrix may cause a weaker bonding of the particles, thus revealing weak points in the filament. rHDPE filaments also have inconsistent diameters that may lead to irregular extrusion and cause uneven layer deposition, resulting in weaker 3D printed parts [53]. Therefore, further research on the rheological characteristics of the rHDPE filament is needed to determine the optimal printing parameters for achieving the highest-quality prints.

### 3.7. Sound Absorption Test

**Figure 13:**
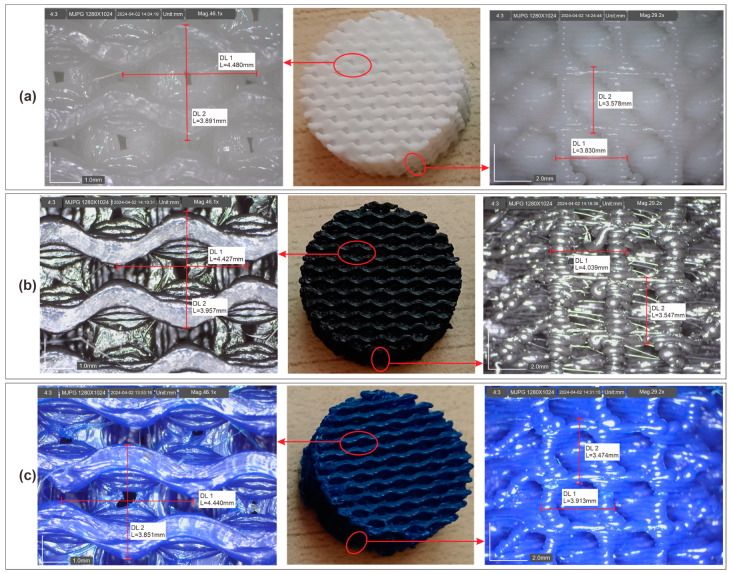
Sound absorption specimens: (**a**) PLA; (**b**) ABS; (**c**) rHDPE + 10%GP.

Three specimens were fabricated using distinct materials: ABS, PLA, and rHDPE + 10%GP. All samples were printed with a consistent layer height of 0.3 mm and an infill density of 50%. The detailed depiction of these specimens is presented in Figure 13. Notably, the cell size of each specimen exhibited remarkable similarity. This measurement ensures that the specimens possess comparable features, facilitating accurate comparison during subsequent sound absorption tests.

**Figure 14:**
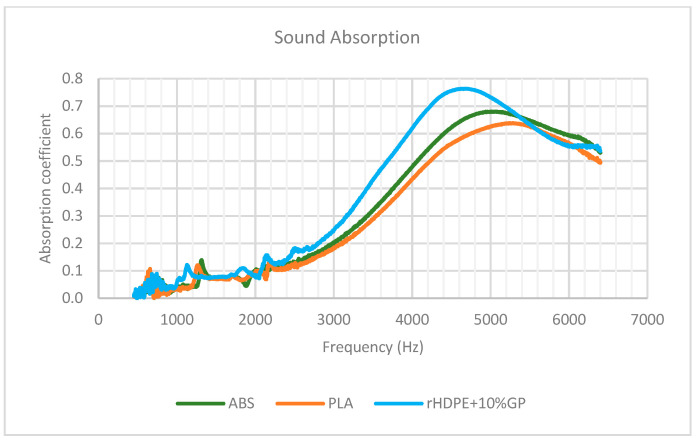
Initial sound absorption test of lattice specimens.

According to the data depicted in Figure 14, the sound absorption coefficient of all samples exhibited an ascending trend with increasing frequency. However, after reaching their respective peak values, the coefficients declined. Specifically for PLA, the maximum absorption occurred at 5360 Hz with coefficients of 0.64. ABS reached its peak at 5200 Hz with coefficients of 0.68, while rHDPE + 10%GP achieved its highest absorption coefficient at 4830 Hz with coefficients of 0.76. It is worth noting that the overall sound absorption coefficient of rHDPE + 10%GP outperformed that of ABS and PLA, particularly within the frequency range of 2160 Hz to 5220 Hz, although the coefficient was lower at frequencies exceeding 5450 Hz.

**Figure 15:**
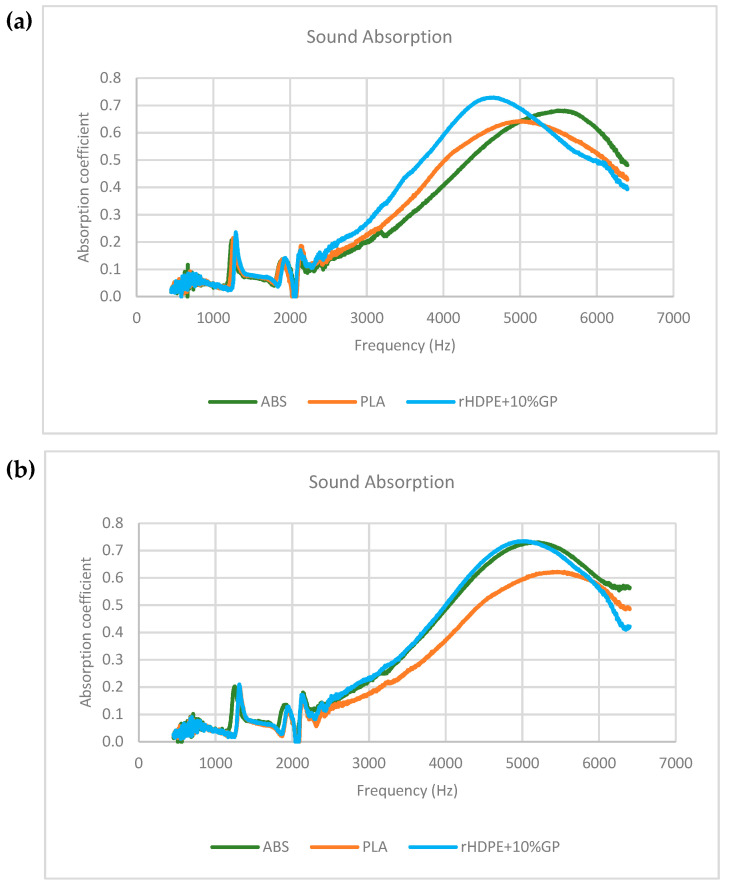
Sound absorption test repetition: (**a**) 2nd test result; (**b**) 3rd test result.

To validate the findings, the test was repeated twice more, and the outcomes are depicted in Figure 15a,b. In Figure 15a, all materials exhibit a consistent trend comparable to the initial test. Conversely, in Figure 15b, the ABS curve nearly aligns with the HDPE curve, albeit slightly lower. The results indicate that the diamond lattice structure of rHDPE + 10%GP is particularly well-suited for absorbing sound waves, exhibiting higher absorption coefficients in the high-frequency ranges when compared to ABS and PLA.

### 3.8. Environmental Implications

Table 12 presents the results of the impact category assessment, showing the amount of environmental impact associated with three distinct waste disposal scenarios: Scenario 1 (incineration), Scenario 2 (landfill disposal), and Scenario 3 (recycling). The choice to compare incineration and landfilling stems from their ubiquity as common end-of-life practices worldwide. The results demonstrate that, across all impact categories, the recycling scenario exhibits lower pollution levels compared to the other two scenarios. Specifically, the recycling system, which transforms bottle caps into 3D filament, yields a remarkable 54% reduction in global warming impact and a 53% reduction in ozone formation impact relative to landfilling (Scenario 2). These reductions are even more pronounced when compared with incineration (Scenario 1), where pollution levels are lower by 99% for global warming and 97% for ozone formation. However, it is essential to note that even Scenario 3, which takes recycling into account, still registers some amount of ionizing radiation, but 29% lower than Scenario 2, which uses landfilling disposal.

**Figure 16:**
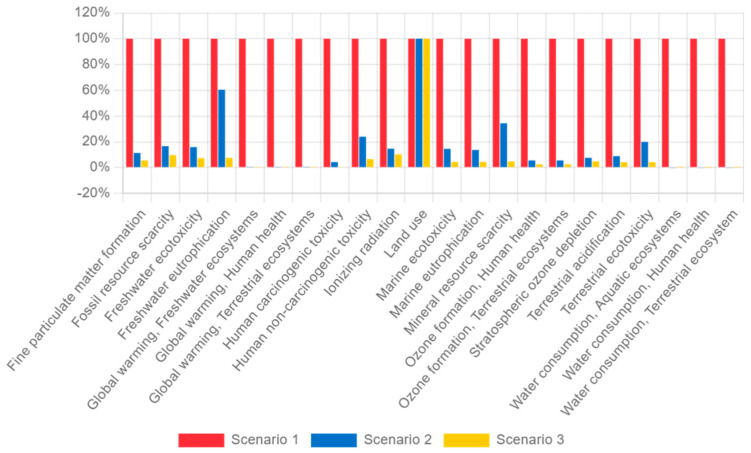
Environmental impact summary.

Figure 16 summarizes the life cycle assessment (LCA) results, highlighting the stark contrast between recycling plastics for 3D printing filament and conventional disposal methods, such as incineration and landfill. Incineration, while capable of generating electricity, exacts a substantial environmental toll due to its energy-intensive nature. The process demands significant energy inputs, including fuel and electricity for equipment operation [54]. However, this advantage is offset by several challenges, notably energy losses and, most critically, the emission of air pollutants and carbon. These emissions occur in substantial quantities, underscoring the environmental impact associated with incineration [55].

Landfilling also carries significant environmental impacts. In landfills, organic waste decomposes anaerobically, leading to the production of greenhouse gases that significantly contribute to global climate change [56]. Inorganic waste such as plastics within landfills undergo slow decomposition, resulting in long-lasting environmental impact and releasing harmful substances to the land [57]. The leachate, a liquid formed when water interacts with solid waste, poses additional risks. It can contain harmful substances, including heavy metals, organic compounds, and pathogens that can contaminate water and soil [58].

In contrast, the recycling scenario by repurposing plastics into 3D printing filament significantly mitigates adverse impacts. The entire process, encompassing the collection, sorting, and processing of recyclables, is inherently more energy-efficient. Recycling also obviates the need to extract and process raw materials, which inherently carry a carbon footprint [59]. By reusing existing materials, natural resources are conserved, and the environmental strain caused by extraction and production is minimized. Additionally, recycling systems exhibit a remarkable ability to minimize harmful pollutant emissions [60]. Compared with incineration and landfilling, recycling significantly lessens the amount of pollutants released into the environment [60].

## 4. Conclusions

In this study, an experiment to recycle HDPE from bottle caps into 3D printing filament was successfully conducted. By optimizing extrusion parameters: setting the temperature at 180 °C, speed at 7 rpm, and incorporating 10% glass powder into the HDPE matrix, a filament with notable tensile strength averaging 25.52 MPa was achieved. To put this into perspective, this tensile strength was slightly higher than that of the ABS filament, which averaged 25.41 MPa, and was only marginally lower than that of the PLA filament, which averaged 28.55 MPa. While the filament exhibited some diameter inconsistencies, it was still usable in 3D printers and capable of producing printed specimens.

Comprehensive mechanical testing provided valuable insights into the performance characteristics of the rHDPE + 10%GP printed specimens in comparison to those made from virgin commercial filaments like ABS and PLA. The results indicated that the rHDPE + 10%GP specimens exhibited lower ultimate tensile, flexural, and compression strengths. Specifically, the average ultimate tensile strength of the rHDPE + 10%GP specimens was 13.94 MPa, which was notably lower than that of ABS at 26.93 MPa and PLA at 28.57 MPa. Similarly, in terms of flexural strength, the rHDPE + 10%GP specimens recorded an average of 41.15 MPa, compared to ABS at 66.13 MPa and PLA at 58.56 MPa. For compression strength, the rHDPE + 10%GP specimens had an average of 22.52 MPa, while ABS and PLA averaged 81.31 MPa and 37.10 MPa, respectively. Additionally, the surface roughness of the rHDPE + 10%GP specimens was observed to be higher, with an average value of 35.28 μm, compared to ABS at 22.43 μm and PLA at 18.17 μm.

However, the rHDPE + 10%GP specimens displayed lower stiffness coupled with a higher strain at break, indicating remarkable ductility and flexibility. This inherent flexibility opens up avenues for applications where high force resistance is not a primary concern, such as in acoustic applications where the ability to absorb sound can be advantageous. A sound absorption test was further carried out to confirm the potential of rHDPE + 10%GP for acoustic applications. The result demonstrated that the rHDPE + 10%GP lattice specimens surpassed both ABS and PLA in terms of absorption coefficient. Specifically, while PLA exhibited a maximum absorption coefficient of 0.64 at 5360 Hz and ABS peaked at 0.68 at 5200 Hz, the rHDPE + 10%GP reached its highest absorption coefficient of 0.76 at 4830 Hz. Importantly, the rHDPE + 10%GP consistently outperformed ABS and PLA, especially within the frequency range of 2160 Hz to 5220 Hz, although its performance tapered off at frequencies exceeding 5450 Hz. This finding underscores the potential of recycled materials like HDPE for use in acoustic applications. Moreover, the sustainability aspect of rHDPE cannot be overlooked. Life cycle analysis comparing recycling with common disposal methods such as landfilling and incineration revealed that recycling significantly reduces pollution levels, global warming impact, and ozone formation.

While rHDPE may not match the mechanical strength of some virgin filaments in printing products, its versatility, flexibility, and sustainability make it a valuable material for diverse applications. As technology evolves and research progresses, we anticipate further enhancements in the quality and performance of recycled materials for 3D printing. Embracing recycled materials not only fosters sustainability but also paves the way for innovation and creativity in 3D printing applications.

## Figures and Tables

**Table 1:** Extrusion parameters.

Input Material	Extrusion Temperature (°C)	Extrusion Speed (RPM)
100% rHDPE	180	5
rHDPE + 10%GP	200	7
rHDPE + 20%GP	220	9

**Table 2:** Design of experiment.

Input Material	Extrusion Temperature (°C)	Extrusion Speed (RPM)
rHDPE	180	5
rHDPE	200	7
rHDPE	220	9
rHDPE + 10%GP	180	7
rHDPE + 10%GP	200	9
rHDPE + 10%GP	220	5
rHDPE + 20%GP	180	9
rHDPE + 20%GP	200	5
rHDPE + 20%GP	220	7

**Table 3:** Printing parameters for ABS mechanical test specimens [36].

Printing Parameter	Value
Extrusion temperature	215 °C
Bed temperature	60 °C
Print speed	90 mm/s
Layer height	0.1 mm
Infill pattern	Linear
Infill percentage	50%
Fan speed	100%
Build plate adhesion	None

**Table 4:** Printing parameters for PLA mechanical test specimens [37].

Printing Parameter	Value
Extrusion temperature	220 °C
Bed temperature	110 C
Print speed	30 mm/s
Layer height	0.5 mm
Infill pattern	Linear
Infill percentage	80%
Fan speed	100%
Build plate adhesion	None

**Table 5:** Printing parameters for rHDPE mechanical test specimens.

Printing Parameter	Value
Extrusion temperature	205 °C
Bed temperature	110 °C
Print speed	80 mm/s
Layer height	0.3 mm
Infill pattern	Linear
Infill percentage	50%
Fan speed	50%
Build plate adhesion	Brim

**Table 6:** Filaments’ tensile test results.

Material	Temperature (°C)	Speed (rpm)	Repetition	UTS (MPa)	Modulus Young (MPa)	%Elongation at Break
rHDPE	180	5	1	20.24	456.95	28.30%
rHDPE	180	5	2	20.31	677.6	12.71%
rHDPE	180	5	3	19.92	756.02	16.34%
rHDPE	200	7	1	20.97	775.56	10.69%
rHDPE	200	7	2	23.20	1023.8	16.97%
rHDPE	200	7	3	22.86	1117	24.08%
rHDPE	220	9	1	22.91	668.21	28.30%
rHDPE	220	9	2	21.08	955.84	13.52%
rHDPE	220	9	3	19.94	732.14	21.54%
rHDPE + 10%GP	180	7	1	23.41	1008.8	23.18%
rHDPE + 10%GP	180	7	2	24.96	754.48	25.74%
rHDPE + 10%GP	180	7	3	28.20	999.51	21.48%
rHDPE + 10%GP	200	9	1	20.71	636.7	22.08%
rHDPE + 10%GP	200	9	2	21.73	685.02	22.08%
rHDPE + 10%GP	200	9	3	20.06	529.06	20.56%
rHDPE + 10%GP	220	5	1	22.24	591.12	23.57%
rHDPE + 10%GP	220	5	2	20.96	568.53	17.54%
rHDPE + 10%GP	220	5	3	16.37	356.18	22.52%
rHDPE + 20%GP	180	9	1	23.60	625.29	27.33%
rHDPE + 20%GP	180	9	2	21.40	682.05	18.55%
rHDPE + 20%GP	180	9	3	20.84	602.25	19.29%
rHDPE + 20%GP	200	5	1	22.65	623.29	23.01%
rHDPE + 20%GP	200	5	2	22.55	707.7	14.34%
rHDPE + 20%GP	200	5	3	20.99	693.84	17.38%
rHDPE + 20%GP	220	7	1	22.64	690.42	22.51%
rHDPE + 20%GP	220	7	2	20.28	647.87	16.75%
rHDPE + 20%GP	220	7	3	18.67	301.9	18.35%
ABS	-	-	1	28.92	1429	4.19%
ABS	-	-	2	23.31	1363.2	12.50%
ABS	-	-	3	23.98	1442.9	5.36%
PLA	-	-	1	29.09	1721	24.52%
PLA	-	-	2	28.38	1844.8	18.67%
PLA	-	-	3	28.18	1882.5	28.30%

**Table 7:** Thermal properties of specimens.

Sample	T_m_ (°C)	$\Delta H_{m}$	X_c_ (%)
rHDPE flakes	133.67	179.79	61.36
rHDPE filament	134.67	166.69	56.89
rHDPE + 10GP filament	134.67	190.53	72.25
rHDPE + 20GP filament	134.67	188.47	80.41

**Table 8:** 3D printed specimens’ tensile test results.

Material	Repetition	UTS (MPa)	Yield Strength (MPa)	Modulus Young (MPa)	%Elongation at Break
ABS	1	27.60	25.13	956	9.94%
	2	26.38	24.14	963.47	10.07%
	3	26.83	25.30	976.32	8.98%
	AVG	26.93	24.86	965.26	9.66%
	STDEV	0.5	0.51	8.39	0.005
PLA	1	28.98	27.60	947.80	6.74%
	2	29.43	27.20	1049.20	6.99%
	3	27.30	26.20	1029.40	7.98%
	AVG	28.57	27.00	1008.80	7.24%
	STDEV	0.91	0.59	43.88	0.01
rHDPE + 10%GP	1	15.86	10.87	310.12	23.63%
	2	15.15	10.37	318.87	18.34%
	3	10.81	6.30	206.44	30.20%
	AVG	13.94	9.18	278.48	24.06%
	STDEV	2.23	2.05	51.06	0.05

AVG: Average, STDEV: Standard deviation.

**Table 9:** 3D printed specimens’ flexural test results.

Material	Repetition	UFS (MPa)	Yield Strength (MPa)	Modulus Flexural (MPa)	%Strain at Break
ABS	1	66.34	53.48	1435.5	21.20%
	2	65.49	50.7	1429.1	21.20%
	3	66.55	51.90	1437.5	21.20%
	AVG	66.13	52.03	1434.03	21.20%
	STDEV	0.46	1.14	3.58	0
PLA	1	62.54	54.3	1739.1	13.70%
	2	56.52	52.3	1807.9	13.69%
	3	56.63	51.60	1860	15.13%
	AVG	58.56	52.73	1802.33	14.18%
	STDEV	2.81	1.14	49.51	0.01
rHDPE + 10%GP	1	37.02	16.67	441.76	24.53%
	2	45.72	20.35	549.08	19.49%
	3	40.72	24.86	485.9	24.89%
	AVG	41.15	20.63	492.25	22.97%
	STDEV	3.56	3.35	44.04	0.02

AVG: Average, STDEV: Standard deviation.

**Table 10:** 3D printed specimens’ compression test results.

Material	Repetition	UCS (MPa)	Yield Strength (MPa)	Elastic Modulus (MPa)	%Strain at Break
ABS	1	81.85	9.73	310.91	69.97%
	2	84.96	9.69	316.05	69.97%
	3	77.12	9.33	317.96	69.97%
	AVG	81.31	9.58	314.97	69.97%
	STDEV	3.22	0.18	2.98	0
PLA	1	34.29	26.8	1172.2	67.48%
	2	38.01	25.8	1162.1	62.73%
	3	39.00	25.30	1157.4	65.60%
	AVG	37.10	25.97	1163.90	65.27%
	STDEV	2.03	0.62	6.17	0.02
rHDPE + 10%GP	1	24.26	7.29	184.97	41.91%
	2	26.15	7.4	211.65	51.91%
	3	17.16	5.90	192.42	50.60%
	AVG	22.52	6.86	196.35	48.14%
	STDEV	3.87	0.68	11.24	0.04

AVG: Average, STDEV: Standard deviation.

**Table 11:** 3D printed specimens’ surface roughness test results.

Material	Ra (μm)						Average Ra (μm)	Standard Deviation
	1	2	3	4	5	6		
ABS	22.267	22.488	22.738	22.45	22.203	22.374	22.429	0.173
PLA	19.461	18.145	17.996	17.217	18.027	17.871	18.169	0.671
rHDPE + 10%GP	34.751	34.709	34.669	34.771	37.508	37.35	35.282	1.276

**Table 12:** Environmental impacts of three scenarios.

Impact Categories	Unit	Scenario 1	Scenario 2	Scenario 3
Fine particulate matter formation	DALY	5.30 × 10^−5^	6.05 × 10^−6^	2.99 × 10^−6^
Fossil resource scarcity	USD2013	5.60505	0.93361	0.54938
Freshwater ecotoxicity	species.yr	2.09 × 10^−12^	3.33 ×10^−13^	1.54 × 10^−13^
Freshwater eutrophication	species.yr	7.39 × 10^−11^	4.47 × 10^−11^	5.62 × 10^−12^
Global warming, freshwater ecosystems	species.yr	2.49 × 10^−10^	6.17 × 10^−13^	2.83 × 10^−13^
Global warming, human health	DALY	0.00302	7.49 × 10^−6^	3.43 × 10^−6^
Global warming, terrestrial ecosystems	species.yr	9.10 × 10^−6^	2.26 × 10^−8^	1.04 × 10^−8^
Human carcinogenic toxicity	DALY	1.98 × 10^−7^	8.62 × 10^−9^	6.40 × 10^−10^
Human non-carcinogenic toxicity	DALY	6.10 × 10^−7^	1.47 × 10^−7^	4.04 × 10^−8^
Ionizing radiation	DALY	2.29 × 10^−8^	3.37 × 10^−9^	2.38 × 10^−9^
Land use	species.yr	2.47 × 10^−14^	2.47 × 10^−14^	2.47 × 10^−14^
Marine ecotoxicity	species.yr	2.12 × 10^−12^	3.10 × 10^−13^	9.45 × 10^−14^
Marine eutrophication	species.yr	1.64 × 10^−12^	2.25 × 10^−13^	7.31 × 10^−14^
Mineral resource scarcity	USD2013	0.00128	0.00044	6.21 × 10^−5^
Ozone formation, human health	DALY	1.81 × 10^−7^	1.01 × 10^−8^	4.76 × 10^−9^
Ozone formation, terrestrial ecosystems	species.yr	2.60 × 10^−8^	1.46 × 10^−9^	6.96 × 10^−10^
Stratospheric ozone depletion	DALY	9.64 × 10^−9^	7.32 × 10^−10^	4.69 × 10^−10^
Terrestrial acidification	species.yr	7.75 × 10^−8^	6.91 × 10^−9^	3.30 × 10^−9^
Terrestrial ecotoxicity	species.yr	1.94 × 10^−10^	3.87 × 10^−11^	8.34 × 10^−12^
Water consumption, aquatic ecosystems	species.yr	1.59 × 10^−12^	−4.03 × 10^−15^	6.05 × 10^−17^
Water consumption, human health	DALY	5.83 × 10^−6^	−1.48 × 10^−8^	2.22 × 10^−10^
Water consumption, terrestrial ecosystem	species.yr	3.54 × 10^−8^	−9.01 × 10^−11^	1.35 × 10^−12^

## Data Availability

Data is available within the article. The data presented in this study are available on request from the corresponding author.
